# Suppression Benefits Boys in Taiwan: The Relation between Gender, Emotional Regulation Strategy, and Mental Health

**DOI:** 10.3389/fpsyg.2017.00135

**Published:** 2017-02-06

**Authors:** Kuang-Hui Yeh, Olwen Bedford, Chih-Wen Wu, Shu-Yi Wang, Nai-Shing Yen

**Affiliations:** ^1^Institute of Ethnology, Academia SinicaTaipei, Taiwan; ^2^Department of Psychology, National Taiwan UniversityTaipei, Taiwan; ^3^Department of Psychology, University of MacauTaipa, Macau; ^4^Department of Psychology, National Chengchi UniversityTaipei, Taiwan

**Keywords:** emotion regulation, gender differences, internalizing problems, negative affect, suppression, reappraisal, Chinese culture, adolescents

## Abstract

Emotion regulation (ER) strategies have a clear impact on mental health outcomes. In 2 studies (*N* = 695, *N* = 433) we investigated gender differences in the use of 2 ER strategies (reappraisal and suppression) to handle parent-child conflict in Taiwanese adolescents. We also identified the implications of these differences for some negative emotions (self-blame and resentment) and internalizing problems (psychosomatic symptoms and social withdrawal). Results of the correlation analyses in both studies indicated that reappraisal and suppression ER strategies are positively correlated only in male Taiwanese adolescents. Hierarchical regression analyses in the second study confirmed that reappraisal buffers male but not female adolescents against the negative effects of suppression on the arousal of negative affect and internalizing problems.

## Introduction

*Emotion regulation* (ER) is the ability to manage emotional reactions to achieve goal-directed outcomes (Cole et al., 1994). It can be conscious or unconscious, automatic or effortful (Gross and Thompson, 2007). Research has established that individuals use a variety of strategies to influence which emotions they experience, and when they experience them (Gross, 1998). However, the various ER strategies have different implications for adaptation and adjustment; some strategies correlate with positive outcomes and others with negative ones (Richards and Gross, 2000). Either way, ER strategies have a clear effect on mental health (Gresham and Gullone, 2012), making them an important topic of investigation.

In this paper, we investigate some gender differences in the use of ER strategies. We focused on adolescents because although ER in adults has received significant empirical attention, few studies have targeted adolescents (Bariola et al., 2011). Adolescence is an important age to investigate because it is a time of transition that encompasses numerous biological, cognitive, and social changes that result in increased negative affect and risk for internalizing symptoms (Arnett, 1999), particularly for girls (Costello et al., 2003). Prior to puberty, boys and girls have the same prevalence of most affective disorders, but during adolescence, the gender ratio increases to a two-to-one female-to-male ratio, the same as in the general adult population (Kessler et al., 1994).

In this study, we investigate the ER strategies of adolescents in Taiwan. We explore whether a gender difference in use of ER strategies exists, and whether this difference, if any, has particular implications for mental health outcomes. In the following, we first review the relevant literature on the process model of emotion regulation, and then introduce important aspects of Chinese culture to develop our hypotheses regarding the relationship between the two prominent ER strategies, and their respective implications for mental health.

### The process model of emotion regulation

Gross's (1998) widely-accepted process model of emotion regulation is premised on the assumption that emotions unfold over time with the implication that ER strategies can be differentiated in terms of when they have their impact. *Antecedent-focused strategies* center on actions prior to activation of an emotional response. For example, *cognitive reappraisal* (hereafter *reappraisal*) involves changing the way one thinks about potentially emotion-eliciting situation (e.g., construing a performance as a learning opportunity rather than a potential for failure) (Gross and John, 2003; Ray et al., 2008). *Response-focused strategies* refer to the behavioral response to an emotion that is already underway. For example, *expressive suppression* (hereafter *suppression*) involves inhibiting an ongoing emotion by controlling or neutralizing emotional behavior (e.g., not crying when sad) (Gross and John, 2003). Most ER strategies can be classified into these two types. As clear representatives of these two types, reappraisal and suppression have received substantial research attention (Bariola et al., 2011).

In two sets of studies of undergraduates, Gross and John (2003) and John and Gross (2004) found that use of reappraisal is associated with healthier long-term affective, cognitive, and social consequences in daily life than suppression. Recent meta-analyses support these findings (Aldao et al., 2010; Webb et al., 2012; Hu et al., 2014): Reappraisal correlates significantly and positively with positive indicators of mental health, and negatively with negative ones; suppression correlates significantly and negatively with positive indicators of mental health, and positively with negative ones, with moderate to strong effect sizes across dozens of studies.

Studies of ER in adolescents have reported similar findings. In their investigations of female North American adolescents, both Lanteigne et al. (2014) and Eastabrook et al. (2014) found that all negative emotions and internalizing behaviors measured in their studies had a significant positive correlation with suppression and a significant negative correlation with reappraisal. Similarly, Betts et al. (2009) found that the Australian adolescents who reported high levels of depressive symptomatology also reported more suppression and less reappraisal as compared to adolescents who reported low levels of depressive symptomatology.

The process model of emotion regulation (John and Gross, 2004) can be used to explain the difference in health outcomes. Reappraisal occurs early, so it can modify the entire emotional sequence before emotion response tendencies have been fully generated. Thus, reappraisal requires few cognitive resources to implement, and produces interpersonal behavior that is appropriately focused on the interaction partner. It is likely to be perceived by the partner as emotionally engaging and responsive. In contrast, suppression comes late in the emotion-generative process, and requires continual effort to manage emotional responses as they arise. This effort consumes cognitive resources that could otherwise be used for optimal performance in the social context. John and Gross also pointed out that suppression may create a sense of discrepancy between inner experience and outer expression. This sense of not being true to oneself may lead to negative feelings about the self and alienate the individual from others, impeding the development of emotionally close relationships and contributing further to interpersonal behavior that is distracted, strained, and avoidant.

### Chinese culture

The explanation provided by the process model of emotion regulation for the impact of suppression on health outcomes is not compatible with findings from Chinese societies. In contrast to Western societies that encourage free and open emotional expression, empirical evidence has demonstrated that collectivistic cultural norms, which support interdependence and social harmony, encourage emotional suppression in circumstances in which there is concern about harming others so as to preserve relationships (Matsumoto et al., 2008). In Chinese societies (which emphasize relational harmony), suppression of emotional responses may be functional because it gives individuals time to identify the proper emotional response that fits the current social context (Uchida et al., 2009). That is, suppression can function in a prosocial manner. Instead of being purely a maladaptive emotion regulation strategy, the social consequences associated with suppression may depend on other factors, such as cultural beliefs or the goals of suppression (Butler et al., 2007). For example, suppressing anger with a friend in order to preserve the relationship may be adaptive.

A number of studies have identified cultural differences in the implications of emotion-expressive behavior and suppression. For example, Butler et al. (2009) found differences between European Americans and Asian Americans in the relation between emotion-expressive behavior and harmful physiological responses. They were inversely related for the European Americans, but positively related for Asian Americans. Soto et al. (2011) found that use of suppression does not correspond to the same harmful effects for Hong Kong Chinese as for European Americans. Furthermore, Hu et al.'s (2014) meta-analysis of ER studies (including publications in Chinese databases) found that although cultural values had no moderating effect on the relationship between reappraisal and mental health indicators, the correlation of suppression with negative indicators of mental health was significantly stronger in the Western samples (*r* = 0.19) than in the Eastern samples (*r* = 0.06), and suppression correlated negatively with positive indicators of mental health (e.g., quality of life) only in the Western samples. Suppression may be less harmful in a Chinese context.

### The relation between suppression and reappraisal

In developing their measures of reappraisal and suppression with American undergraduates, Gross and John (2003) found the scales “to be independent in each sample (mean *r* = −0.01)”, indicating that “individuals who frequently use reappraisal were no more or less likely to use suppression than individuals who use reappraisal infrequently” (p. 351). A series of CFA confirmed that the independence model, two factors correlating zero, was the best fit. In discussing these findings, Gross and John (2003) referred to their participants as “reappraisers” or “suppressors.” That is, they implied a trait perspective by classifying participants into one type or the other, although in a subsequent review they did acknowledge that there is “considerable room for change, especially over long periods of time” (John and Gross, 2004, p. 1321).

The results of more recent research suggest a different perspective. For example, Hu et al. (2014, p. 355) concluded from their meta-analysis that it is likely that “individuals do not adopt only one of the two strategies. Instead, people employ a combination or use both strategies at different times.” Eftekhari et al.'s (2009) study of ER and mental health outcomes in American undergraduate women provides evidence to support this contention. They identified four patterns of use of reappraisal and suppression: High on both; low on both; high reappraisal and low in suppression; and moderate reappraisal, low suppression. Consistent with past research, the group that relied mainly on reappraisal had the best mental health outcomes. However, the largest cluster identified in their sample was comprised of women who were high on both reappraisal and suppression; they used both equally. The implication of these studies is that it may be important to understand the pattern of ER strategies used instead of emphasizing use of a particular strategy. With this perspective, it is less important to emphasize which strategy is best, and more important to understand how the strategies interact, and how they relate to mental health outcomes. Neither of these studies reported the correlation between reappraisal and suppression in their samples.

However, Matsumoto et al. (2008) specifically examined the correlation between reappraisal and suppression from a cross-cultural perspective. Although they noted that Gross and John (2003) had identified an orthogonal relationship in American undergraduates, they expected that this finding would not hold true in other cultural contexts. In fact, their analysis of data from undergraduates in 23 countries generally confirmed their hypothesis that cultures high on individualism have a negative relationship between suppression and reappraisal, and cultures high in social order, hierarchy, and long-term orientation (which includes Chinese societies) have a positive relationship between suppression and reappraisal. They also found that countries high in social order tended to have higher scores on suppression.

Given Matsumoto et al.'s (2008) findings, we expect that the relationship between suppression and reappraisal is positive in Taiwanese adolescents because of an initial suppression followed by subsequent reappraisal (or the reverse) allowing individuals to select the proper emotional expression to preserve social order and maintain interpersonal relationships.

Hypothesis 1: Reappraisal and suppression ER strategies are positively correlated in Taiwanese adolescents.

As Gross and John's (2003) analysis of four samples of North American undergraduates indicated no relationship between suppression and reappraisal, and recent studies of North American female adolescents reported a significant negative correlation [(Eastabrook et al., 2014) (*r* = −0.19, *p* < 0.05); Lanteigne et al., 2014 (*r* = −0.31, *p* < 0.05)], confirmation of Hypothesis 1 would also constitute preliminary evidence for a cultural difference in the pattern of use of these ER strategies.

### Gender, suppression, and mental health outcomes

Expressing emotions is generally viewed as unmanly in a wide variety of cultures (Brody, 2000). Parents report teaching sons greater emotional control than daughters, and boys report that they are expected to inhibit their emotional expression to a greater extent than girls (Underwood et al., 1992). Men tend to use suppression more than women do (Gross and John, 2003). As we have noted, regulating emotion through suppression is associated with affective disorders and problem behaviors, especially internalizing ones (Nolen-Hoeksema and Aldao, 2011). However, women are three times more likely than men to be diagnosed with a major depressive disorder (Maciejewski et al., 2001; Hyde et al., 2008). They also tend to brood more often than men in response to a stressful event, which results in a greater lifetime prevalence of comorbid depression and anxiety (Gorman, 2006). How is it that men use suppression as a regulatory strategy to a greater degree than women, yet are less susceptible to affective disorders?

We propose that gender differences in emotional responding may arise—at least in part—from differences in the pattern of use of ER strategies that stem from physiological differences. Whittle et al. (2011) found that the brain regions women use to regulate negative emotion are more associated with emotional processing, while those men use are more associated with cognitive processing. They posited that the mechanism underlying increased reactivity to negative stimuli in women involves their sustained limbic activity as compared to men. Although men initially respond similarly to women, they recover faster because their regulatory mechanisms are engaged more quickly to dampen affective reactions (Williams et al., 2005). Therefore, it is reasonable to infer that if it takes some effort to switch strategies between different categories (such as suppression and reappraisal), men would be able to do it more easily than women. In the context of Chinese culture, which supports suppression in some contexts, the greater ease of switching may make it more likely for men than women to use both strategies together.

Hypothesis 2: The correlation between suppression and reappraisal ER strategies is stronger for male than female Taiwanese adolescents.

We expect that this difference in regulatory patterns makes male adolescents less vulnerable to negative emotions and internalizing behaviors than female adolescents. In line with our earlier observation that it may be less important to emphasize which strategy is best, and more important to understand how the strategies interact, and how they relate to mental health outcomes, we propose the following hypothesis:

Hypothesis 3: The gender difference in the relation of suppression and reappraisal, including their interaction, corresponds to lower scores on affective problems for male than female adolescents.

We conducted two studies to investigate whether suppression of emotions by Taiwanese adolescents corresponds to a different mental health impact by gender via the relationship between suppression and reappraisal. We examined the first two hypotheses in Study 1, and collected a fresh data set to examine Hypothesis 3 in Study 2.

## Study 1

In Study 1, we investigated the relationship between reappraisal and suppression in Taiwanese adolescents. We administered the emotion regulation questionnaire (Gross and John, 2003) to a large sample of junior high students in Taiwan. We calculated the Pearson product moment correlation to examine the strength of the relationship between reappraisal and suppression, and then we calculated the correlations separately for the male and female participants in each sample. We expected that (H1) reappraisal would be positively related to suppression, and that (H2) the relationship would be stronger for males as compared to females.

### Method

#### Participants and procedures

The study was approved by the institutional review board of National Taiwan University. After providing informed written consent, participants were ensured anonymity and presented questionnaires in class, which took about 5 min to complete. No payments or incentives were provided. We obtained a valid sample of 695 students (348 females and 347 males; mean age 14.05, *SD* = 0.35) from nine junior high schools throughout northern, central, and southern Taiwan. Descriptive statistics for the sample are summarized in Table 1.

**Table 1:** **Descriptive statistics and the correlation between reappraisal and suppression in Studies 1 and 2**.

	**Reappraisal**		**Suppression**		**Correlation *(r)* between reappraisal and suppression**	**Gender difference of correlation *(z)***
	***M***	***SD***	***M***	***SD***		
**STUDY 1**						
Total (N = 695)	3.19	1.15	2.60	1.30	0.20^**^	
Male (*n* = 347)	3.19	1.19	2.50	1.34	0.30^**^	2.88^**^
Female (*n* = 348)	3.20	1.12	2.69	1.27	0.09	
**STUDY 2**						
Total (*N* = 433)	3.19	1.47	2.65	1.30	0.18^**^	
Male (*n* = 204)	3.19	1.19	2.61	1.31	0.32^**^	2.97^**^
Female (*n* = 229)	3.19	1.11	2.68	1.29	0.05	

*There was no significant difference by gender in use of reappraisal or suppression*.

** *p < 0.01*.

#### Measures

##### Emotion regulation questionnaire

We used the Chinese version (Liu et al., 2015) of Gross and John's (2003) 10-item emotion regulation questionnaire (ERQ), which measures adolescents' use of emotion regulation strategies in the last 6 months. The items are rated on a 6-point Likert scale from 0 (*strongly disagree*) to 5 (*strongly agree*). The questionnaire consists of two subscales: Reappraisal (6 items; e.g., When I'm faced with a stressful situation, I make myself think about it in a way that helps me stay calm) and Suppression (4 items; e.g., I control my emotions by not expressing them). Subscale scores were calculated by averaging participants' summed scores on subscale items. High scores indicate a greater inclination to use a specific emotion regulation strategy. For this study, the Cronbach's alphas were 0.88 for reappraisal and 0.77 for suppression.

### Results

Reappraisal correlated significantly with suppression (*r* = 0.20, *p* < 0.01; see Table 1), suggesting that increased use of one emotional coping strategy corresponded to increased use of the other. H1 is supported. H2, that the correlation between reappraisal and suppression would be stronger for males (*r* = 0.30, *p* < 0.01) than for females (*r* = 0.09, *p* = 0.09), was also supported. We used moderated regression with suppression as the dependent variable, and gender, reappraisal, and their interaction as predictors. The results showed the model reached significance (*F* = 13.91, *p* < 0.01), and the interaction effect was also significant (β = 0.15, *p* < 0.01), indicating that the relation between reappraisal and suppression for males was different from that for females. We used Fisher's transformation to obtain the *z* values and found a significant difference in the correlation coefficients between the males and females (*z* = 2.88, *p* < 0.01).

### Brief discussion

We expected and found an overall positive correlation between suppression and reappraisal. However, although the boys exhibited a significant positive correlation, the girls' correlation showed only a marginal positive association, which was statistically smaller than the *r* = 0.3 for boys. Contrary to the Western samples previously reviewed (where there was either a null or negative association for both males and females), for all Taiwanese adolescents, there was a positive association (albeit a stronger one for males).

## Study 2

In Study 2, we sought to replicate the findings from Study 1 via another adolescent sample as well as to broaden it by focusing on the impact of the pattern of use of reappraisal and suppression on the psychological adjustment of each gender. To focus the domain of psychological adjustment under examination, we constrained the context to parent-adolescent conflict. We selected this area because for many adolescents, parent-child conflict is the primary source of stress in their daily life (Chan, 1998) and it affects their emotional well-being (Shek, 1998). Past research has established that negative emotional arousal due to parent-child conflict can mediate the effect of that conflict on adolescents' internalizing problem behaviors (Yeh, 2011). This finding clearly implies that if (in the context of parent-adolescent conflict) adolescents can lessen their negative emotions by efficiently regulating them, then the negative impact of parent-adolescent conflict on adolescents' problem behaviors is reduced.

In order to examine whether it is possible that suppression and reappraisal interact to reduce negative emotions, which in turn correspond to reduced problem behavior, we asked adolescents about their negative emotional arousal, and their frequency of use of suppression and reappraisal to regulate their negative emotions when in conflict with their parents. We selected resentment and self-blame as the target negative emotions, and psychosomatic symptoms and social withdrawal as the target internalizing problems because a strong association between these negative emotions and these internalizing problems has been demonstrated for Taiwanese adolescents in conflict with their parents (Yeh, 2011).

We conducted hierarchical regression analyses to evaluate the main and interaction effects of the two ER strategies and gender variables on adolescents' psychological adjustment. Three outcomes are expected. First, (as in Study 1) we expect to find an overall positive correlation between suppression and reappraisal, and a stronger relationship between reappraisal and suppression for males than females. Second, consistent with the literature, we expect reappraisal will have a positive main effect on psychological outcomes, whereas suppression will exert a negative impact. Third, due to the cultural value placed on suppression in some contexts, and the physiological gender difference in regulation of negative emotions, we expect that the negative impact of high suppression will be buffered by high reappraisal only in males.

### Method

#### Participants and procedures

We collected a new valid sample of 433 students (204 males and 229 females) in grade 7 (average age = 14.66, *SD* = 0.72) from four junior high schools in Taiwan. With the consent of their teachers and parents, they completed a 30-min self-report questionnaire with separate father and mother sections, which measured the negative emotions that arose in conflict with either of the parents, the strategies frequently used to regulate their negative emotions, and their extent of internalizing problem behaviors during the last 6 months. The questionnaire was presented in a counterbalanced order (father-mother or mother-father). Participation was voluntary and confidential. Approximately 73 percent of participants lived with both parents and all with at least one parent. The average age of fathers and mothers was 44.82 years (*SD* = 4.85) and 41.56 years (*SD* = 4.66), respectively. The majority of parents (74% fathers and 63% mothers) were middle-class technicians and service or sales workers. Slightly over a quarter (28%) of the mothers were housewives.

#### Measures

##### Emotion regulation questionnaire

Adolescents' use of ER strategies was measured with the ERQ as in Study 1, but we asked the participants to respond while considering their experiences of conflict with their parents. For this study, the Cronbach's alphas were 0.88 for reappraisal and 0.77 for suppression.

##### Negative emotion arousal

We assessed negative emotional arousal by asking participants about their negative feelings or experiences arising from conflict with their parents in the past 6 months. We used Yeh's (2011) two subscales to measure the two types of conflict-related emotion: resentment and self-blame. We presented each item separately for the father and the mother. Participants rated each item on a 6-point Likert scale ranging from 0 (*strongly disagree*) to 5 (*strongly agree*), with higher scores reflecting greater emotional intensity in that given emotion. We examined resentment with 14 items asking participants to rate their feelings of unfairness, being ignored, revenge, or feeling mortified during conflict. Example items are: When I get into conflict with my father/mother, “I feel mortified” and “I curse or talk back silently.” For this study, Cronbach's alphas were 0.92 for both father and mother versions. Self-blame was defined in terms of character, as proposed by Janoff-Bulman (1979). The adolescents were instructed to assess their self-attributions that arose after the conflict and their enduring feelings of guilt associated with the conflict. The subscale consists of nine items. Example items are: After getting into conflict with my father/mother, “I feel regret” and “I feel disappointed in myself.” The father and mother versions had Cronbach's alphas of 0.95 and 0.94, respectively.

##### Internalizing problem behavior

We used two self-report scales, psychosomatic symptoms and social withdrawal, to examine internalizing problem behaviors. Psychosomatic symptoms were assessed through a 17-item scale adapted from the Somatic Complaints, Thought Problems, and Attention Problems subscales of the Chinese version (Leung et al., 2006) of the Child Behavior Checklist–Youth Self-Report Form (CBCL-YSR) for ages 11–18 (Achenbach, 1991). Social withdrawal was assessed using a 10-item scale. The items evaluated the degree to which adolescents disengage from others or the environment, such as refusing to talk with others or being secretive, passive, dispirited, or timid. They were selected from the Chinese version (Leung et al., 2006) of the Withdrawn subscale of the CBCL-YSR (Achenbach, 1991) and the Withdrawal/Timidity subscale of the Adolescent Social Behavior Scale (Hung, 1997). Participants rated the frequency of the specific problem behavior on a 6-point scale ranging from 0 (*never*) to 5 (*always*). Scale scores were determined by summing participants' ratings on scale items. For this study, Cronbach's alpha for the psychosomatic symptoms scale was 0.92 and for Social withdrawal it was 0.86.

### Results

As in Study 1, we found a significant positive correlation between reappraisal and suppression for the sample (*r* = 0.18, *p* < 0.01; see Table 1). For boys, the correlation was 0.32 (*p* < 0.01) and for girls it was 0.05 (*p* = 0.49). Also as in Study 1, the boys had a significantly stronger correlation than the girls (*z* = 2.97, *p* < 0.01).

Because all correlations between the two types of conflict-related emotion in the father and mother versions were highly significant (resentment in both versions, *r* = 0.72; self-blame in both versions, *r* = 0.76), scores on the specific emotion in the father and mother versions were combined for subsequent analyses. Cronbach's alphas for the summed scores were 0.93 for resentment and 0.96 for self-blame.

#### Gender differences in strategies, negative emotion, and internalizing behavior

We calculated the means, standard deviations, and correlations for all major variables separately by gender (see Table 2). As expected, female participants scored significantly higher than males on both negative emotions: resentment [female, *M* = 2.19, *SD* = 1.14; male, *M* = 1.77, *SD* = 1.04; *t*_(441)_ = 4.08, *p* < 0.01] and self-blame [female, *M* = 2.16, *SD* = 1.33; male, *M* = 1.64, *SD* = 1.33; *t*_(441)_ = 4.06, *p* < 0.01]. Female participants also reported higher levels of both internalizing problem behaviors, with significantly higher scores on psychosomatic symptoms [female, *M* = 1.63, *SD* = 1.14; male, *M* = 1.32, *SD* = 0.92; *t*_(441)_ = 3.09, *p* < 0.01] and social withdrawal [female, *M* = 1.79, *SD* = 1.04; male, *M* = 1.56, *SD* = 0.99; *t*_(441)_ = 2.37, *p* = 0.02].

**Table 2:** **Study 2: Correlations, means, and standard deviations of the main variables**.

	**1**	**2**	**3**	**4**	**5**	**6**	***M***	***SD***
1. Reappraisal		0.05	−0.05	0.15^*^	−0.13^*^	−0.23^**^	3.19	1.11
2. Suppression	0.32^**^		0.42^**^	0.27^**^	0.56^**^	0.65^**^	2.68	1.29
3. Resentment	0.05	0.30^**^		0.46^**^	0.59^**^	0.56^**^	2.19	1.14
4. Self-blame	0.15^*^	0.21^**^	0.43^**^		0.32^**^	0.28^**^	2.16	1.33
5. Psychosomatic symptoms	−0.01	0.36^**^	0.59^**^	0.39^**^		0.66^**^	1.63	1.14
6. Social withdrawal	−0.06	0.52^**^	0.52^**^	0.31^**^	0.58^**^		1.79	1.04
*M*	3.19	2.61	1.77	1.64	1.32	1.56		
*SD*	1.19	1.31	1.04	1.33	0.92	0.99		

*Numbers below the diagonal are from male data (n = 204); those above are from female data (n = 229)*.

* *p < 0.05*,

** *p < 0.01*.

#### Correlations between ER strategy and dependent variables

At the bivariate level, the correlations between suppression and the dependent variables were all significant for both genders. Specifically, suppression was significantly associated with resentment (*r* = 0.30 and *r* = 0.42 for males and females, respectively; both *p*s < 0.01), self-blame (*r* = 0.21 and *r* = 0.27 for males and females, respectively; both *p*s < 0.01), psychosomatic symptoms (*r* = 0.36 and *r* = 0.56 for males and females, respectively; both *p*s < 0.01), and social withdrawal (*r* = 0.52 and *r* = 0.65 for males and females, respectively; both *p*s < 0.01), indicating that the more frequently adolescents used suppression to regulate their negative emotions, the more likely they were to experience negative emotional arousal and internalizing problem behaviors when in conflict with their parents.

In contrast, the correlation patterns between reappraisal and the dependent variables did differ by gender. Unexpectedly, reappraisal was positively related to self-blame for both genders (*r* = 0.15 for males and females; both *p*s < 0.05), and not related to resentment for either. However, only in female participants did we find the expected significant negative relationship between reappraisal and the internalizing problems: psychosomatic symptoms (*r* = −0.13, *p* < 0.05) and social withdrawal (*r* = −0.23, *p* < 0.01). In other words, regardless of gender, adolescents who tended to employ reappraisal to cope with their emotions demonstrated higher levels of self-blame, but the use of reappraisal only correlated with a decrease in internalizing problem behaviors for female adolescents.

All the dependent variables correlated significantly with one another. For both genders, resentment significantly correlated with self-blame, psychosomatic symptoms, and social withdrawal (males, *r* = 0.43, 0.59, and 0.52; females, *r* = 0.46, 0.59, and 0.56, respectively; all *p*s < 0.01). Self-blame correlated with psychosomatic symptoms and social withdrawal (males, *r* = 0.39 and 0.31; females, *r* = 0.32 and 0.28, respectively, all *p*s < 0.01). Psychosomatic symptoms and social withdrawal correlated with each other (males, *r* = 0.58, females, *r* = 0.66, both *p*s < 0.01).

#### Hierarchical regression analyses

We conducted hierarchical regression analyses to test whether reappraisal interacted with suppression to influence the dependent variables for males but not females. Demographic variables included parents' education levels, whether the child lives with one or both parents, and family structure [nuclear, stem (one married child living with parents or grandparents), or extended (siblings and their spouses living with parents or grandparents)]. These variables were entered into Block 1 in the hierarchical analyses as control variables. The two ER strategies (reappraisal and suppression) and gender and their interaction term were entered into Block 2 to test their main and interacting effects on negative emotional arousal and internalizing problem behaviors. The four full models (including the main, two-way, and three-way interaction terms together) all reached significant levels (*F* = 8.49, 5.45, 15.15, and 28.93 for the models with the dependent variable set to resentment, self-blame, psychosomatic symptoms, and social withdrawal, respectively, all *p*s < 0.01).

While the main effect of gender was not significant (β = −0.06, *p* = 0.11) on social withdrawal, it was significant on resentment (β = −0.16, *p* < 0.01), self-blame (β = −0.15, *p* < 0.01), and psychosomatic symptoms (β = −0.11, *p* < 0.01), revealing that female adolescents are more vulnerable to negative emotional arousal and internalizing problems than male adolescents are. In addition, the four three-way interaction effects (Reappraisal × Suppression × Gender) all reached significant levels (on resentment, β = −0.14; on self-blame, β = −0.18; on psychosomatic symptoms, β = −0.15; and on social withdrawal, β = −0.14; all *p*s < 0.05). Therefore, we conducted the following analyses separately with the male and female data.

#### Negative emotional arousal and internalizing problems

The results for negative emotional arousal and internalizing problems for male and female participants are summarized in Tables 3, 4.

**Table 3:** **Study 2: Betas from hierarchical regression analyses for ER strategies and their interaction predicting male dependent variables**.

	**Resentment**		**Self-blame**		**Psychosomatic symptoms**			**Social withdrawal**		
**Predictors**	**Block 1**	**Block 2**	**Block 1**	**Block 2**	**Block 1**	**Block 2**	**Block 3**	**Block 1**	**Block 2**	**Block 3**
**DEMOGRAPHIC CONTROL VARIABLES**										
Living with single parent vs both parents	0.00	−0.03	−0.04	−0.07	0.04	0.00	0.02	0.04	−0.02	−0.01
**FAMILY STRUCTURE**										
Stem vs Nuclear family	0.01	−0.02	0.09	0.08	0.10	0.07	0.06	0.08	0.03	0.03
Extended vs Nuclear family	−0.10	−0.08	−0.01	−0.03	−0.11	−0.08	−0.03	−0.10	−0.05	−0.02
Father's education	0.10	0.07	0.13	0.08	0.12	0.09	0.05	0.06	0.02	−0.01
Mother's education	−0.06	−0.07	−0.10	−0.12	0.02	0.01	0.06	−0.11	−0.13^*^	−0.10
**EMOTION REGULATION**										
Reappraisal		−0.10		0.05		−0.20^**^	−0.16^**^		−0.29^**^	−0.26^**^
Suppression		0.31^**^		0.17^*^		0.38^**^	0.21^**^		0.57^**^	0.46^**^
Reappraisal × suppression		−0.16^*^		−0.22^**^		−0.21^**^	−0.11		−0.30^**^	−0.24^**^
**NEGATIVE EMOTION AROUSAL**										
Resentment							0.45^**^			0.32^**^
Self-blame							0.16^*^			0.05
*F*	0.76	3.79^**^	0.90	3.27^**^	1.72	6.74^**^	16.53^**^	1.09	16.33^**^	19.45^**^
Δ*R*^2^	0.02	0.12^**^	0.02	0.10^**^	0.04	0.18^**^	0.25^**^	0.03	0.37^**^	0.10^**^
Δ*df*	5/198	3/195	5/198	3/195	5/198	3/195	2/193	5/198	3/195	2/193

*N = 204*.

* *p < 0.05*,

** *p < 0.01*.

**Table 4:** **Study 2: Betas from hierarchical regression analyses for ER strategies and their interaction predicting female dependent variables**.

	**Resentment**		**Self-blame**		**Psychosomatic symptoms**			**Social withdrawal**		
**Predictors**	**Block 1**	**Block 2**	**Block 1**	**Block 2**	**Block 1**	**Block 2**	**Block 3**	**Block 1**	**Block 2**	**Block 3**
**DEMOGRAPHIC CONTROL VARIABLES**										
Living with single parent vs both parents	0.11	0.08	0.00	−0.02	0.12	0.08	0.05	0.03	−0.02	−0.04
**FAMILY STRUCTURE**										
Stem vs Nuclear family	−0.05	0.01	0.01	0.03	−0.12	−0.04	−0.04	−0.16^*^	−0.06	−0.06
Extended vs Nuclear family	0.09	0.08	0.09	0.10	0.00	−0.02	−0.05	0.08	0.05	0.02
Father's education	0.12	0.18^*^	−0.12	−0.11	0.00	0.06	0.00	−0.06	0.01	−0.04
Mother's education	−0.13	−0.13	0.03	0.02	−0.07	−0.06	−0.01	−0.02	0.00	0.04
**EMOTION REGULATION**										
Reappraisal		−0.08		0.16^*^		−0.16^**^	−0.14^**^		−0.25^**^	−0.23^**^
Suppression		0.42^**^		0.26^**^		0.55^**^	0.37^**^		0.66^**^	0.52^**^
Reappraisal × suppression		0.05		0.02		0.01	−0.01		−0.05	−0.06
**NEGATIVE EMOTION AROUSAL**										
Resentment							0.39^**^			0.32^**^
Self-blame							0.06			0.02
*F*	1.61	7.22^**^	0.97	3.60^**^	1.65	14.40^**^	20.69^**^	2.02	27.00^**^	30.65^**^
Δ*R*^2^	0.04	0.17^**^	0.02	0.10^**^	0.04	0.31^**^	0.14^**^	0.04	0.45^**^	0.09^**^
Δ*df*	5/223	3/220	5/223	3/220	5/223	3/220	2/218	5/223	3/220	2/218

*N = 229*.

* *p < 0.05*,

** *p < 0.01*.

##### Resentment and self-blame

The influence of the two ER strategies and their interaction on resentment and self-blame took different paths by gender. With demographic variables controlled, suppression exerted positive main effects on resentment, and self-blame for both genders, whereas reappraisal was only significantly associated with self-blame in females. The more inclined participants were to suppress their emotions when in conflict with their parents, the more frequently they reported feelings of resentment and self-blame. Female participants who used reappraisal on a regular basis also experienced greater self-blame as compared to those who did not use it frequently.

For males, the reappraisal × suppression interaction term significantly predicted both resentment and self-blame, suggesting that reappraisal moderates the relationship between suppression and these negative emotions. However, for females, the reappraisal × suppression interaction was not significantly associated with resentment or self-blame.

**Figure 1:**
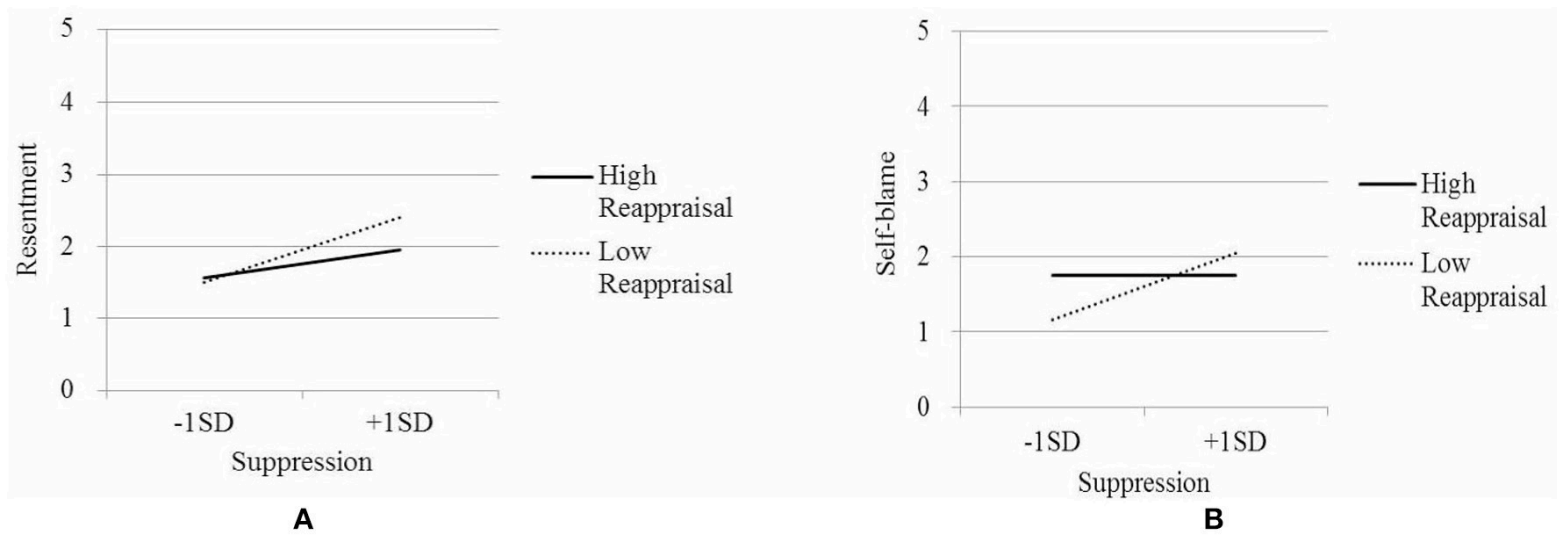
**Interaction effects of ER strategies on negative emotion arousal for males**.

Figure 1A shows that reappraisal buffered male adolescents against the negative effects of suppression on the arousal of resentment in parent-child conflict (simple slope with high reappraisal: β = 0.19, *p* = 0.04; simple slope with low reappraisal: β = 0.44, *p* < 0.01). Figure 1B shows the disordinal interaction effects of reappraisal and suppression on self-blame (simple slope with high reappraisal: β = 0.00, *p* = 0.99; simple slope with low reappraisal: β = 0.34, *p* < 0.01). In both figures, when suppression was used on a regular basis, male adolescents who also frequently adopted a reappraisal strategy demonstrated better outcomes in terms of resentment and self-blame as compared to those who mainly relied on suppression with little use of reappraisal.

##### Psychosomatic symptoms and social withdrawal

In Block 2 with demographic variables controlled, for both genders, reappraisal had negative main effects on psychosomatic symptoms and social withdrawal, while suppression had significant positive effects on them. The more frequently adolescents used reappraisal to regulate their emotions, the less likely they were to report internalizing problem behaviors, and the reverse was true for suppression. Notably, the reappraisal × suppression interaction term was statistically significant in predicting psychosomatic symptoms and social withdrawal only for males, suggesting that the impact of suppression depends on the level of reappraisal for males.

**Figure 2:**
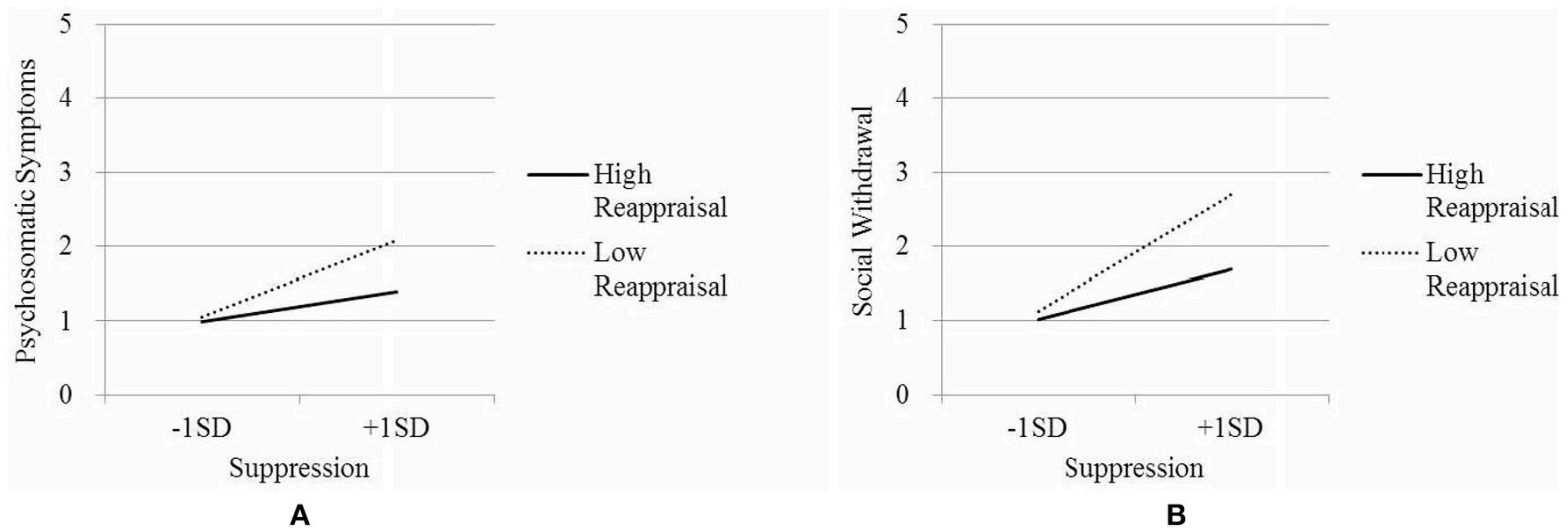
**Interaction effects of ER strategies on internalizing problem behaviors for males**.

Figures 2A,B show the interaction effects of reappraisal and suppression on psychosomatic symptoms (simple slope of suppression with high and low reappraisal, respectively: β = 0.22, *p* = 0.02; β = 0.55, *p* < 0.01) and social withdrawal (β = 0.34 and β = 0.79, *p*s < 0.01, for the simple slope of suppression with high and low reappraisal, respectively). For both indicators, male adolescents who habitually used reappraisal to deal with their emotions experienced decreased levels of internalizing problem behaviors as compared to those who used reappraisal infrequently, regardless of their level of suppression. However, the difference between high and low reappraisers on internalizing problems was larger for those who were also suppressors; that is to say, high reappraisal appeared to protect male adolescents from the impact of high suppression on internalizing problem behaviors. Hypothesis 3 is confirmed.

## General discussion

We examined three hypotheses in two studies: (1) whether the relation between reappraisal and suppression in Taiwan is different from previously reported results for North American societies, (2) whether the relation between reappraisal and suppression differs by gender in Taiwan, and (3) whether this difference corresponds to lower scores for affective problems for males than females.

### The relation of reappraisal and suppression

The results of both of our studies confirmed an overall positive relation between reappraisal and suppression among Taiwanese adolescents, supporting Matsumoto et al.'s (2008) contention that a positive relationship between reappraisal and suppression should correspond to an overall cultural emphasis on emotional restraint as a whole (as found in Chinese societies). Our results also provided evidence of a cultural difference in the relation of reappraisal and suppression in adolescents in comparison to the negative correlations between these ER strategies identified in studies of North American adolescents (e.g., Lanteigne et al., 2014; Eastabrook et al., 2014).

Both of our studies also confirmed the stronger positive correlation between the two ER strategies for boys than girls. To our knowledge, no previous study has examined gender differences in the correlation of ER strategies in adolescents. Although previous studies have noted that in many cultures expressing emotions is viewed as unmanly (Brody, 2000), and that men tend to use suppression more than women do (Gross and John, 2003), we could find no studies examining gender differences in the relation of reappraisal and suppression, or how gender differences in their patterns of interaction relate to mental health outcomes. Given the pervasiveness of differences in gender norms and physiology with regard to emotional expression, we expect that most other societies would also exhibit gender differences in the pattern of interaction of reappraisal and suppression with implications for mental health outcomes.

### The impact of ER strategies

As in past studies of adults (e.g., Aldao et al., 2010; Webb et al., 2012; Hu et al., 2014), our female participants scored higher than the males on measures of negative affect and internalizing problems. They also experienced higher levels of resentment and self-blame when in conflict with their parents, as well as more psychosomatic symptoms, and social withdrawal in daily life. Also in accordance with the literature showing suppression is associated with negative consequences on the individual level (e.g., John and Gross, 2004), we found that suppression correlated positively with negative affect and internalizing behaviors for both genders.

However, our finding stands in contrast to more recent cross-cultural studies that found suppression may require less physiological effort for Asians than European Americans (Butler et al., 2009), and thus may have a reduced negative or null association with negative mental health outcomes such as low life satisfaction and depression (Soto et al., 2011). We expect that it may be important to differentiate purposeful or functional suppression strategies (such as forbearance) from non-purposeful or dysfunctional suppression strategies, meaning that it is necessary to take a processing perspective when discussing the impact of this strategy in Chinese societies (and other societies that emphasize interpersonal harmony). In this study, we did not differentiate different types of suppression strategies with the result that we found a positive correlation between suppression and negative affect and internalizing behaviors for both genders. The main effect may represent a dysfunctional suppression strategy, and the interaction may represent a functional suppression strategy.

Our results for reappraisal differed from the consensus in the literature. Past studies identified a negative correlation between reappraisal and negative affect. In contrast, we found that reappraisal corresponded positively with self-blame for both genders, and there was no relation to resentment (instead of a negative one) for either gender. The past literature has also identified a negative relationship between reappraisal and internalizing behavior. For the boys in our study, reappraisal had no relationship with either internalizing behavior. Girls did have the expected negative relation between reappraisal and social withdrawal, although the relation with psychosomatic symptoms was not significant (instead of negative). In other words, the beneficial effect of reappraisal was not stable, and even led to negative outcomes.

Our results reflect a proposal highlighted by Aldao et al. (2010). They suggested that the effectiveness of supposedly adaptive strategies may be more context-dependent than the detrimental effects of maladaptive strategies. For example, problem-solving may be adaptive only when one is facing a solvable problem. Likewise, there may be situations in which reappraisal is not the most adaptive strategy. In contrast, maladaptive strategies such as suppression may have detrimental effects in a wide range of circumstances (see Nolen-Hoeksema et al., 2008). Alternatively, adaptive strategies may be primarily related to positive affect and well-being, which is not necessarily the opposite, or absence, of negative affect (e.g., Watson et al., 1988). In this same vein, we note that the selection of a particular strategy may be context dependent. For example, Chinese adolescents adopt different emotional display rules with parents and peers (Wang et al., 2012), which suggests that ER strategies for managing conflict with parents may differ from those adopted when interacting with peers.

### Gender differences

We investigated gender differences in patterns of ER strategies and delineated possible paths through which these patterns contribute to vulnerability to affective symptoms. Our findings suggest that although both genders use comparable levels of suppression and reappraisal strategies, Taiwanese male adolescents use suppression and reappraisal jointly more than female adolescents do. We propose that Taiwanese male adolescents' use of suppression goes beyond repressing or constraining behavior to become a tactical way to handle stressful interpersonal situations harmoniously. Thus, Taiwanese male adolescents' initial suppression of emotional reactions may be habitual in order to allow them to fit into a given social context. After that, their use of reappraisal or other cognitive strategies allows them to regulate their negative emotions efficiently and in turn reduce their vulnerability to affective symptoms. Another possibility is that they initially reappraise the conflict situation and then follow with suppression of their emotional reaction in order to maintain interpersonal harmony.

Despite sharing the same social context, Taiwanese female adolescents are biologically primed to appraise stressors as more severe (Hyde et al., 2008). As females they are more likely to use emotion-focused strategies, and so require more effort to regulate their emotions than men do (Williams et al., 2005). These factors predispose them to separate suppression and reappraisal, and to hold negative emotions somewhat longer than boys, which in turn contributes to a higher level of vulnerability to affective symptoms as compared to males. Thus, both genders may use a comparable degree of suppression and reappraisal in daily life, but differences in regulatory patterns lead to divergent mental health profiles in terms of the rate of affective symptoms.

In addition to the biologically-based difference in processing is the possibility that the general cultural endorsement of emotional suppression to preserve harmony in relationships that we posited would impact both genders, has a much greater impact on boys via masculinity norms. Chinese conceptions of masculinity particularly emphasize restraint and self-control (Bedford and Hwang, 2011), which may manifest as forbearance. By practicing restraint and forbearance, boys may be enacting Chinese principles of masculinity through suppression. Future research might investigate the relation of the practice of forbearance to the use of ER strategies and mental health outcomes in Chinese societies, with particular attention to gender differences.

We also note an interesting finding related to resentment. Tables 3, 4 show that across both genders, resentment is a pretty strong predictor of adolescents' psychosomatic symptoms and social withdrawal when controlling for the effects of demographic variables and regulation strategies. In previous studies, researchers have not paid enough attention to resentment, and they have failed to link it with adolescents' problem behaviors in the parent-child conflict context. This issue deserves more attention in the future.

### Limitations and future directions

There are some limitations to the present study. First is the self-report nature of the data, and, as with all survey studies, the problem of common method variance and social desirability response bias. These can influence participants' responses to the investigated variables, and thus may limit the predictive power of the results. Future research should include non-questionnaire measures.

Second, the use of cross-sectional data and the absence of experimental manipulation precluded assessment of causality or causal direction of the relationship between the regulation strategies and problem behaviors. In fact, there could be causality in both directions. Previous findings suggest that adolescents with psychological symptoms are prone to a negative interpretation of their parents' behavior, thereby increasing their negative emotion arousal or increasing use of dysfunctional strategies when conflicting with parents (Chan, 1998). Further, an adolescent's personality might be a third variable contributing to both negative emotions and emotion regulation in response to parent–adolescent conflict and internalizing problems. These issues should be investigated in future research.

Finally, we suggest that effective ER changes arousal and its associated systems and processes as required by the perceived opportunities and demands of the situation. As a result, emotion–cognition interaction is a major factor in virtually all processes that affect the emotion regulation of adolescents. A single strategy is far from sufficient to judge the effectiveness of regulation or its association with personal adaptation or maladaptation. A pattern encompassing different strategies and underlying motivations would be an appropriate target for future study.

## Author contributions

KY and OB: Substantial contributions to the conception or design of the work and analysis and interpretation of data, revising it critically for important intellectual content, final approval of the version to be published, agreement to be accountable for all aspects of the work in ensuring that questions related to the accuracy or integrity of any part of the work are appropriately investigated and resolved. CW, SW, and NY: Substantial contributions to the acquisition and analysis of data, initial drafting of the work, final approval of the version to be published, agreement to be accountable for all aspects of the work in ensuring that questions related to the accuracy or integrity of any part of the work are appropriately investigated and resolved.

## Funding

This work was supported by two grants from the Ministry of Science and Technology, Taiwan, Republic of China (Study 1: NSC 96-2752-H-001-001-PAE; Study 2: NSC 97-2410-H-001-082-SS3).

### Conflict of interest statement

The authors declare that the research was conducted in the absence of any commercial or financial relationships that could be construed as a potential conflict of interest.
