# Supplementary material for: Suppression Benefits Boys in Taiwan: The Relation between Gender, Emotional Regulation Strategy, and Mental Health
Source: Front Psychol. 2017 Feb 6;8:135. doi: 10.3389/fpsyg.2017.00135 (PMC5292407; doi:10.3389/fpsyg.2017.00135)
Supplement: Supplementary file 1 [file Table1.docx]

**Appendix A**

**Internalizing Problem Behavior Measures (6-point scales)**

Psychosomatic Symptoms Scale Items

1. I feel confused when I deal with things.

2. I suffer from insomnia or have trouble sleeping.

3. I have nightmares.

4. I feel tired of doing everything.

5. I have trouble concentrating.

6. I overeat or under eat because of tension.

7. I repeat certain acts over and over.

8. I feel discouraged.

9. I get hurt a lot easily.

10. I cry at intervals.

11. I feel giddy and dizzy.

12. I ache all over.

13. I have allergy attacks easily.

14. I am nauseated or feel sick for unknown reasons.

15. I have migraines or headaches for unknown reasons.

16. I have difficulty breathing or chest tightness.

17. I get sudden cramps.

Social Withdrawal Scale Items

1. I refuse to talk with others.

2. I keep my thoughts to myself.

3. I don’t get along with others.

4. I suffer alone rather than ask others for help.

5. I have no interest in others.

6. I speak with a quiet or slow voice.

7. I don’t express my feelings.

8. I am frustrated with my relationships.

9. I have no energy in crowds.

10. I don’t get involved with others.
